# An Uncommon Anatomical Variation: Passage of the Ovarian Artery Through a Fenestrated Ovarian Vein in an Elderly Female Cadaver

**DOI:** 10.7759/cureus.85700

**Published:** 2025-06-10

**Authors:** Marisa Knott, Mykyta Lyashenko, Justin Nguyen, Samantha Thurman, Codi Vernace, Matthew Wofford, Uzochukwu Adabanya, Matthew D Overturf

**Affiliations:** 1 Medical School, Edward Via College of Osteopathic Medicine, Monroe, USA; 2 Anatomical Sciences, Edward Via College of Osteopathic Medicine, Monroe, USA

**Keywords:** anatomical variation, cadaveric case report, embryological development, fenestrated ovarian vein, ovarian artery

## Abstract

Ovarian vein fenestration is an extremely rare vascular anomaly characterized by the temporary splitting and rejoining of the ovarian vein. Unlike duplication, fenestration involves a single vein dividing into multiple channels before converging again; it is often discovered incidentally during imaging or surgical procedures. This case report highlights a rare anatomical variant involving the ovarian artery and vein observed in an elderly female cadaver. During the routine dissection of a 93-year-old female's abdominopelvic region, we observed that the left ovarian artery passed through a fenestrated segment of the left ovarian vein. Although there have been few reports of fenestrations in the left ovarian vein, the traversing of such a fenestration by the ovarian artery remains unprecedented. This anatomical peculiarity has significant clinical implications, particularly in pelvic congestion syndrome, interventional radiology, and gynecologic surgery, due to its potential to complicate diagnosis and procedural outcomes.

## Introduction

Anatomical variations of the venous system are commonly encountered and hold considerable clinical significance, particularly during surgical procedures and diagnostic imaging. Among these variations is venous fenestration, a rare vascular anomaly in which a single vein splits into two channels before reuniting. This has been documented in several prominent veins, including those in the cerebral and iliac systems. However, ovarian vein fenestration remains exceedingly rare and underreported in the literature, with only one published case to date [1].

The ovarian veins develop from the subcardinal veins during embryonic formation, with the right ovarian vein typically draining into the inferior vena cava and the left into the left renal vein [2]. Deviations from this standard arrangement can arise from incomplete regression, aberrant anastomosis, or the persistence of embryonic venous channels [3]. Anatomical studies affirm that these variations, which include duplications and atypical terminations, are often asymptomatic but can complicate procedures such as gonadal vein embolization and surgeries in urology and gynecology [4]. Misidentifying such variants may result in misdiagnosis or unexpected intraoperative bleeding.

In this case report, we describe a novel finding observed in a cadaver, where the left ovarian artery was seen traversing a hiatus in the corresponding ovarian vein, indicating an uncommon anatomical course. There have been prior attempts to categorize such variations, which, while comprehensive, lack standardized criteria and do not encompass all reported variability [5,6]. To our knowledge, the variation documented in this study has not been previously reported, spotlighting a significant gap in existing classification systems and emphasizing the need for ongoing updates in anatomical documentation to better inform surgical practices and educational frameworks.

## Case presentation

**Figure 1:**
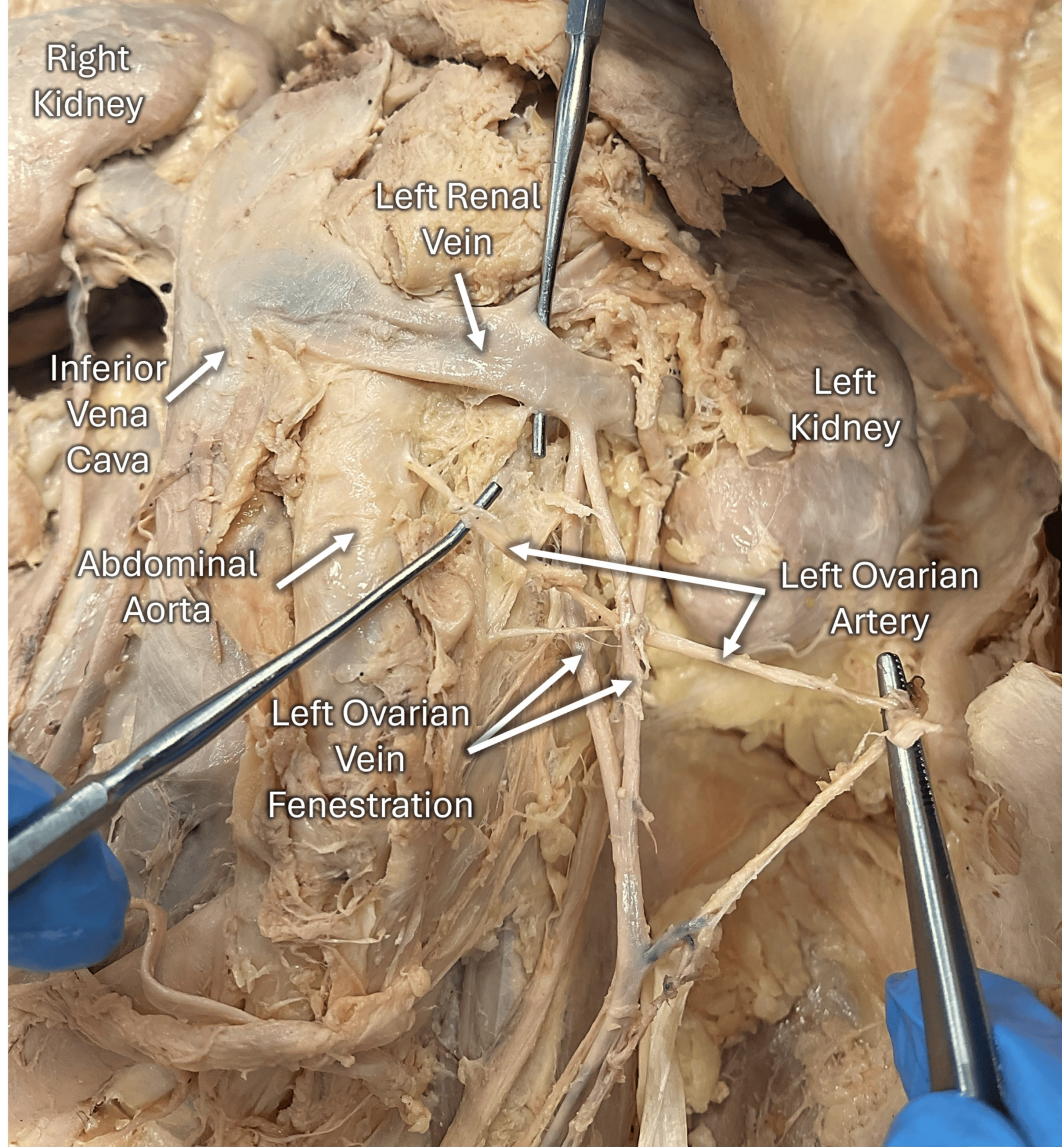
Left ovarian artery passing through a fenestrated left ovarian vein

During the abdominopelvic dissection of a 93-year-old female cadaver, the left ovarian artery was noted to pass through a fenestrated left ovarian vein, located proximal to the left renal vein (Figure 1). In the right pelvic region, the ovarian vasculature appeared typical, showing no recognizable anatomical variations. Examination of the pelvic cavity indicated that the cadaver had undergone a hysterectomy and bilateral oophorectomy, with the ovarian vessels ligated distally. No further abnormalities or deviations in the ovarian structure or vasculature were identified.

## Discussion

Ovarian vein fenestration is a rare anatomical variant that remains unaddressed, mainly in the medical literature. The only published case documents a fenestration of the left ovarian vein observed during cadaveric dissection, where no structures traversed the fenestrated segment and no associated pathology was noted [1]. This anomaly differs from duplication, which involves the formation and persistence of two distinct venous trunks, whereas fenestration suggests a temporary bifurcation that eventually rejoins. Although it is likely asymptomatic in most instances, this variant could have significant implications in specific clinical scenarios.

From an embryological perspective, fenestrations may arise due to incomplete fusion or the persistence of segments within the subcardinal venous system, which is responsible for developing the gonadal veins [3]. Variations in the regression or anastomosis of these vessels can lead to complex venous architectures. Documented cases of unilateral duplication and atypical terminations of ovarian veins in cadaveric dissections further emphasize the prevalence of such anomalies, even in the absence of specific reports on fenestration [4].

Clinically, unrecognized ovarian vein variants, including fenestrations, could pose challenges for catheter-based interventions such as ovarian vein embolization for pelvic congestion syndrome (PCS), a condition marked by chronic pelvic pain and dilated gonadal veins. Anomalous venous patterns may also complicate pelvic or retroperitoneal surgeries, increasing the risk of iatrogenic injury or hemorrhage. A fenestrated vein might be misinterpreted on imaging studies as a thrombus, dissection, or variceal formation, particularly in contrast-enhanced computed tomography (CT) or magnetic resonance venography (MRV), if not accurately identified by the radiologist [7].

Given the scarcity of reported cases, it remains uncertain whether ovarian vein fenestration is genuinely rare or simply underrecognized. Routine imaging may fail to detect such variants unless specifically reviewed, and most anatomical studies lack comprehensive mapping of venous bifurcations. Therefore, further cadaveric studies and retrospective imaging analyses are necessary to determine the prevalence and clinical significance of this anomaly.

## Conclusions

Ovarian vein fenestration is a rare and often underreported anatomical variant that carries potential clinical significance. Although it is likely asymptomatic in most cases, this condition can complicate surgical planning, interventional procedures such as embolization, and radiological interpretations, particularly concerning pelvic venous disorders. From an embryological perspective, this anomaly may result from incomplete fusion or the persistence of segments of the subcardinal venous system, aligning with patterns seen in other venous fenestrations.

The sparse documentation, largely limited to a single case report, underscores both the rarity of this condition and the diagnostic oversights that may accompany it. As advancements in medical imaging and intervention techniques continue, a more comprehensive understanding and recognition of such venous variants are crucial. Further cadaveric research and retrospective imaging studies are needed to determine the actual prevalence of ovarian vein fenestration, elucidate its clinical implications, and enhance procedural safety and diagnostic accuracy.
